# *Wolbachia* Infect Ovaries in the Course of Their Maturation: Last Minute Passengers and Priority Travellers?

**DOI:** 10.1371/journal.pone.0094577

**Published:** 2014-04-10

**Authors:** Lise-Marie Genty, Didier Bouchon, Maryline Raimond, Joanne Bertaux

**Affiliations:** Equipe Ecologie Evolution Symbiose, Laboratoire Ecologie et Biologie des Interactions, UMR CNRS 7267, Université de Poitiers, Poitiers, France; Centro de Pesquisas René Rachou, Brazil

## Abstract

*Wolbachia* are widespread endosymbiotic bacteria of arthropods and nematodes. Studies on such models suggest that *Wolbachia*'s remarkable aptitude to infect offspring may rely on a re-infection of ovaries from somatic tissues instead of direct cellular segregation between oogonia and oocytes. In the terrestrial isopod *Armadillidium vulgare*, *Wolbachia* are vertically transmitted to the host offspring, even though ovary cells are cyclically renewed. Using Fluorescence *in situ* hybridization (FISH), we showed that the proportion of infected oocytes increased in the course of ovary and oocyte maturation, starting with 31.5% of infected oocytes only. At the end of ovary maturation, this proportion reached 87.6% for the most mature oocytes, which is close to the known transmission rate to offspring. This enrichment can be explained by a secondary acquisition of the bacteria by oocytes (*Wolbachia* can be seen as last minute passengers) and/or by a preferential selection of oocytes infected with *Wolbachia* (as priority travellers).

## Introduction

*Wolbachia pipientis* are endosymbiotic bacteria present in many arthropods and nematodes, which manipulate host reproduction to enhance maternal transmission. The success of this transmission to the progeny partly relies on oocyte infection from the stem cell stage, the oogonia. A low *Wolbachia* density in these stem cells would result in a stochastic loss of the infection [1]. To secure this position, a strategy for *Wolbachia* would be to reach it early and in sufficient numbers. However *Wolbachia* do not always reach germline precursor cells during embryonic development. In *Drosophila* species, distinct *Wolbachia* strains differ in their localization in embryos [2]. Most of the *Wolbachia* strains concentrate in the posterior pole, which contains the germline precursor cells. However, in some cases *Wolbachia* are mainly localized at the anterior pole, in somatic line precursor cells. These distributions remain constant throughout embryogenesis from the early stage (preblastoderm) to the late stage (late gastrulation) [3], [2] suggesting that there is no movement or preferential cell division to concentrate *Wolbachia* in the germline precursor cells.

This applies especially to *D. mauritiana*, whose germline precursor cells are poorly infected by *Wolbachia* during embryogenesis [2]. In *D. mauritiana* adults, *Wolbachia* colonize the ovary germline only in limited amounts, whereas *Wolbachia* consistently infect the Somatic Stem Cell Niche (SSCN) [4] or both the Somatic and the Germline Stem Cell Niches (GSCN) [5]. Indeed, in *Drosophila* adult ovarioles, Germline and Somatic Stem Cells are found in “niches” (GSCN and SSCN) mainly consisting of somatic supporting cells that seldom divide. These somatic Niches are envisioned as *Wolbachia* accumulators or reservoirs to sustain or even rescue the ovary infection in adults [4]. Even in *Drosophila* species in which *Wolbachia* are more concentrated in the germline precursor cells during embryonic development, the colonisation of the somatic Niches in adults is conserved (Niche tropism preference being in relation with *Wolbachia* strains rather than with the host genetic background), and supplements the initial embryonic stock of *Wolbachia* [4], [6]. Additionally, in *D. melanogaster* and the Heminoptera *Zyginida pullula*, *Wolbachia* are transmitted to germline cells through bacteriocyte like cells probably of somatic origin, found in the ovaries [7]. So, targeting germline precursor cells during embryonic development is not the only way to infect progeny tissues and an efficient transmission could well rely on somatic tissues by permitting secondary germline infection in adults.

This can even lead us to question the efficiency *Wolbachia*, even if present in the oogonia (the germline stem cells), will deploy to segregate equally enough into daughter cells after mitosis both to maintain an infection pool in the oogonia, which will continue to divide, and to colonize all the oocytes they produce. In early *Drosophila* embryogenesis, *Wolbachia* segregate equally in each daughter cell [3], but their segregation becomes asymmetric in late embryogenesis at least in neuroblast cells, which may result in *Wolbachia* being irregularly distributed in adult tissues [8]. Furthermore, Albertson et al. [8] demonstrated that in neuroblast cells, *Wolbachia* segregation is bound to the cells' asymmetric division pattern. Germline stem cells also divide asymmetrically [9], and while *Wolbachia* are observed in both daughter cells (the future oocyte and the remaining oogonia) [10], there is no indication that the partitioning is balanced. This could result in the stochastic loss of *Wolbachia* in either daughter cells, although this may be hard to spot if *Wolbachia* loss was immediately rescued by secondary infection from somatic Niches. For *Drosophila* species with highly infected germlines, the extent of the *Wolbachia* infection rescue provided by the GSCN and SSCN was recently explored by Toomey et al. [6]. *Wolbachia* densities in the region containing the oogonia are higher when only the adjacent GSCN is colonised, while the enrichment of lowly infected germline cells is posterior to the oogonia stage when only the SSCN is colonised.

Strikingly, in *Brugia malayi*, *Wolbachia* segregation is asymmetric at the beginning of embryogenesis, which leads them to concentrate in specific cell lineages [11]. Even if *Wolbachia* are found in the germline lineage in early embryogenesis [11], they are lost during development [12]. Consequently, the first stages of ovary development lack *Wolbachia*. Accordingly, *Wolbachia* infection in adult germline stem cells requires invasion from neighbouring somatic cells during the larval stages [13]. Therefore in this case it is demonstrated that *Wolbachia*'s remarkable aptitude to infect offspring does not rely on their capacity to directly infect oogonia but instead on an efficient somatic rescue of infection in ovaries.

In the present study, we report a novel case of incomplete *Wolbachia* infection in ovaries. The terrestrial isopod *Armadillidium vulgare* ovary development depends on the reproductive cycle. We demonstrate that adult immature ovaries presented many uninfected areas, with a proportion of infected oocytes as low as 31.5%. This proportion increased in the course of ovary maturation to reach 87.6% for the most mature oocytes, which is close to the known transmission rate to the progeny (82%, [14]). The infection pattern strongly suggests that many oocytes were infected secondarily, but we found no indication of a reservoir directly in the germarium. Our study reinforces the emerging concept that to ensure their vertical transmission, *Wolbachia* may rely on somatic cells or tissues rather than direct cellular segregation.

## Material and Methods

### Ethics statements

All experimental procedures and animal manipulations did not require an ethics statement.

### Animals

*Armadillidium vulgar*e (Crustacean, Isopoda) were reared in the laboratory at 20°C at the natural photoperiod of Poitiers (France), with *ad libitum* food (carrots and dead leaves). We used two lineages of *A. vulgare* infected by the *Wolbachia w*VulC strain [15] which were originally sampled from Celles-sur-Belle (France) and Helsingör (Denmark). The *w*VulC infection was stable through generations since 1991. Ovaries were collected over four years and analysed with Fluorescence *in situ* Hybridization (FISH) to investigate *Wolbachia* infection pattern. To assess *Wolbachia* infection dynamics a random sample of 13 ovaries was collected in 2009 and 2010 from one-year-old females during their first reproduction period and was imaged as described below. As a negative control, eight ovaries collected from females belonging to uninfected lineages originally sampled from Nice (France) and Helsingör (Denmark). Their negative infection status is inferred from an equilibrated sex-ratio as opposed to feminized lineages [16], and an absence of *Wolbachia* detection by PCR on gonads or whole animal [17].

**Figure 1:**
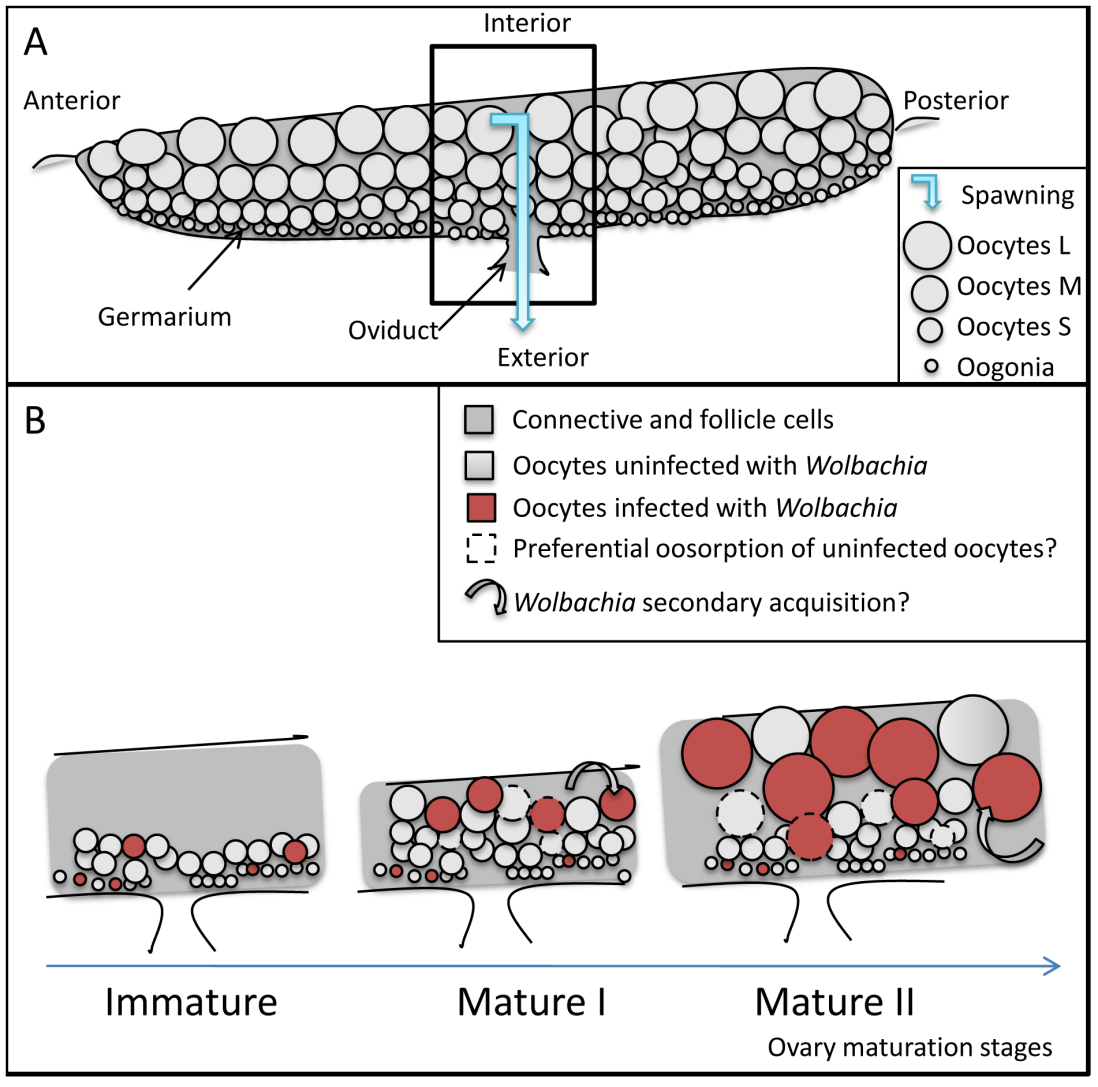
Isopod ovary organisation (A) and inferred infection dynamics (B). **A**: The germarium is composed of oogonia that mature and increase in diameter as they move away from the germarium in the course of ovary maturation The oocytes are encased within two sheets of follicle cells and a layer of connective tissue (dark gray). At spawning muscles compress the ovary to expel the oocytes through the oviduct, which is on the germarium side. **B**: We propose two non exclusive hypotheses could explain *Wolbachia* enrichment in the course of oocyte and ovary maturation (Immature, Mature I, Mature II stages): oocyte selection (oocytes uninfected by *Wolbachia* are preferentially destroyed by oosorption) and/or maturing oocytes acquire *Wolbachia* secondarily from somatic tissues through follicle cells.

Isopod ovaries (described in Besse [18] and Souty-Grosset [19]) are flattened tubular sacks where oocytes are not aligned with their precursor oogonia in separated ovarioles (Fig. 1A). The stem cells of the oocytes and of the follicle cells are both located in the germarium. The germarium is not apical but runs either as a continuous thin line along the side, or as several foci along the margin. Isopod ovaries show a panoistic-like conformation: Oogonia develop into oocytes only, no nurse cells, *i.e*. a follicle just contains one oocyte. The germarium continuously produces a cohort of previtellogenic oocytes (20–110 μm diameters in *Porcellio dilatatus*, [18]) throughout the ovarian cycle, which supplies the cohort of maturing oocytes. These oocytes enlarge as they acquire vitellogenin progressively, first endogenously (diameter 110–250 μm), then exogenously (diameter >250 μm).

### Fluorescent labellings and imaging

Ovaries were dissected and wrapped loosely in aluminium foil to avoid damaging them while pipetting the solutions. The resulting rolls were immersed for 1 h at 4°C in 3% paraformaldehyde-PBS (137 mMNaCl, 8 mM Na_2_HPO_4_,12H_2_O, 1.5 mM KH_2_PO_4_, 3 mMKCl, pH 7.3)-0.1% Triton X-100 for fixation. They were subsequently washed twice in PBS for 15 min and stored at −20°C in 1∶1 (v/v) ethanol-PBS.

The FISH protocol was modified from Manz et al. [20]. Each wrapped ovary was dehydrated in 50, 80, 96% ethanol for 3 min each, then immersed in 100 μl of the following hybridization buffer: 0.9 M NaCl, 35% formamide, 20 mMTris-HCl (pH 8), 0.01% Triton X-100, 10 μL of a 30 ng/μL equimolar mixture of probes W1, 2-Cy3 targeting the 16S rRNA of *Wolbachia* [21], and 10 μL FITC-phalloidin (Sigma, dried from methanol 100 μg.mL^−1^ stock solution). They were hybridized for 1.5 h at 46°C, then washed for 15 min at 48°C in 200 μl of a buffer containing 20 mM Tris-HCl (pH 8), 70 mM NaCl, 5 mM EDTA (pH 8) and 0.01% Triton X-100. The rolls were gently unwrapped in a drop of bidistilled water on a slide to rinse the ovaries and arrange them. After drying they were embedded in CitiDAPI (DAPI 10 μg.mL^−1^; Citifluor AF1 antifading, Citifluor, England). Ovaries were imaged with the Olympus confocal laser scanning microscope described in Chevalier et al. [22] or with an Axio observer A1 (Zeiss) microscope coupled with an Apotome, equipped with a 20X/0.8 plan Apochromatic objective, a 63X/1.25 plan Neofluar oil objective, specific filters for FITC, Cy3 and DAPI (Zeiss Filter Sets 44, 43 and 49), and the Axiovision software (Zeiss). Images were analysed with Image J 1.42q (available at http://rsb.info.nih.gov/ij; developed by Wayne Rasband, National Institute of Health, Bethesda, MD).

The absence of *Wolbachia* FISH labelling in the oocytes of uninfected females was re-assessed in a sub-sampling of four infected and four uninfected females that were treated as described above, in tandem from fixation to imaging. The ovaries were fixed in parallel in the same fixation buffer. During the hybridization steps the samples were alternated uninfected/infected, and they were all hybridized with the same buffer-and-probes mixtures. Phalloïdin labelling was not performed. For imaging, again, we alternated uninfected/infected samples.

In *Wolbachia* infected individuals, to assess *Wolbachia* infection dynamics we counted the number of infected oocytes in ovaries scanned across the whole length but with a 10 μm gap between slices. Each oocyte was tagged, measured (mean diameter out of three measurements) and attributed with an infection status according to the density of *Wolbachia* estimated by eye (uninfected, poorly infected or infected). To control the validity of this sampling, we scanned random locations of the ovaries with a 0.7 μm step. We thus extracted 115 full-depth oocyte scans from four ovaries out of thirteen. The presence of *Wolbachia* and the oocyte diameter were recorded as above to cross-check the previous procedure.

### Statistical procedures

All statistical analyses were performing using R software. Cohort modelling of oocyte diameters was fitted to mixture of normal distributions using a maximum likelihood estimation (mixdist package, e.g. J. Du, unpublished data). Levels of infection by *Wolbachia* in oocytes depending on both the maturation stage of ovaries and the cohort diameter of oocytes were assessed by a multinomial logistic regression. Absence of multicollinearity was checked using the variance-inflation factors in ordinary least square models taking independently into account the maturation stage of ovaries and the cohort diameter of oocytes.

## Results

### Determination of oocyte and ovary categories

We enumerated and measured the mean diameter of oocytes in ovaries dissected from the 13 randomly sampled females. The maximum likelihood estimation of mixtures of statistical distributions (*P*<0.05) based on oocyte diameters and frequencies allowed us to categorize the oocytes into three cohorts: Small (**S**) previtellogenic oocytes, Medium size (**M**) and Large (**L**) vitellogenic oocytes, possibly corresponding to endogenous and exogenous stages of vitellogenesis; large oocytes will be the cohort that are spawned at the next oviposition.

**Figure 2:**
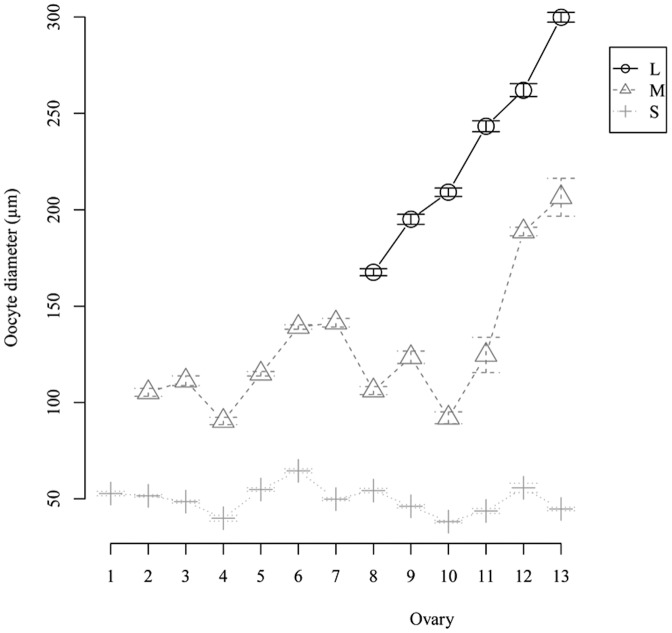
Oocyte cohorts (mean diameter ± s.e.) and their repartition in each ovary. Depending on their maturation stage, ovaries present one to three oocyte cohorts corresponding to oocyte developmental categories called Small (S), Medium (M) and Large (L). According to these categories and the cohorts' mean diameter, we ranked the 13 ovaries along a maturation scale. The sampling broke down into three ovary maturation stages: Immature (with only S oocytes), Mature I (with S and M oocytes) and Mature II (with S, M and L oocytes).

The oocyte distributions also allowed us to rank the ovaries depending on their maturation stages, reconstructing the ovarian cycle (Fig. 2). Based on the number of oocyte cohorts present in an ovary, we determined three maturation stages: Immature (containing only S oocytes, n = 1), Mature I (S and M oocytes, n = 6), Mature II (S, M and L oocytes, n = 6) ovaries. While the previtellogenic oocytes (S) had quite similar mean diameter distributions along maturation, those of vitellogenic oocytes (M and L) were markedly different for each ovary, shifting toward larger sizes as the oocytes matured.

### Fluorescence observations of *Wolbachia* infection

#### Variation of oocyte numbers along ovary maturation is mainly due to a decrease of uninfected oocytes

**Figure 3:**
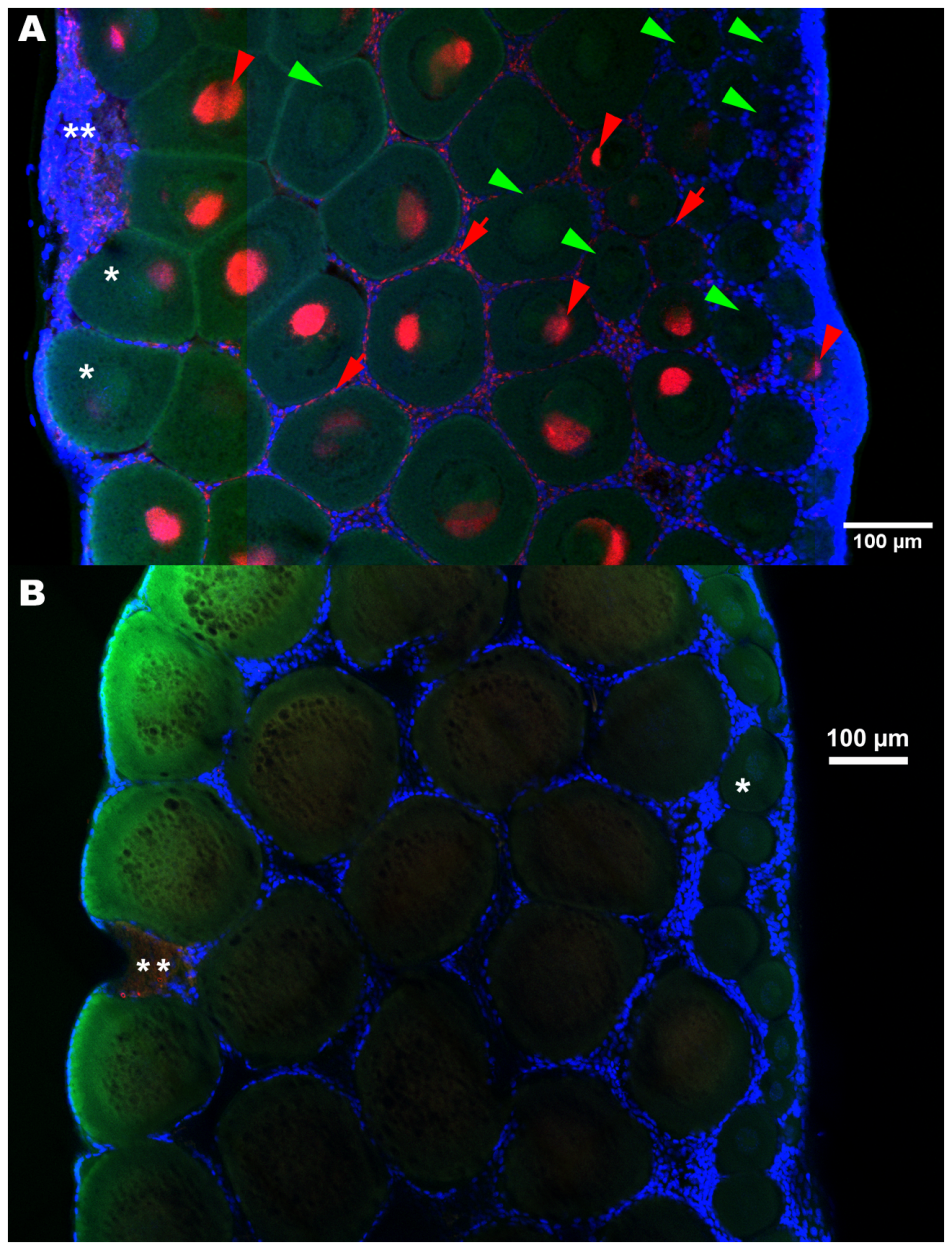
FISH detection of *Wolbachia* in ovaries, infected (A) versus control, uninfected (B). The germaria are on the right borders, areas undergoing degradation on the maturing side of the ovaries (**). Oocyte nuclei are sometimes visible as dots (meiotic prophase) (*). **A**: The *Wolbachia* were present in oocytes of all sizes (red arrowheads) and their follicle cells (red arrows), though many oocytes remained uninfected (green arrowheads), especially the smaller ones. **B**: In uninfected ovaries. Red: *Wolbachia* FISH probe W1,2-Cy3, green: phalloidin, blue: DAPI.

**Figure 4:**
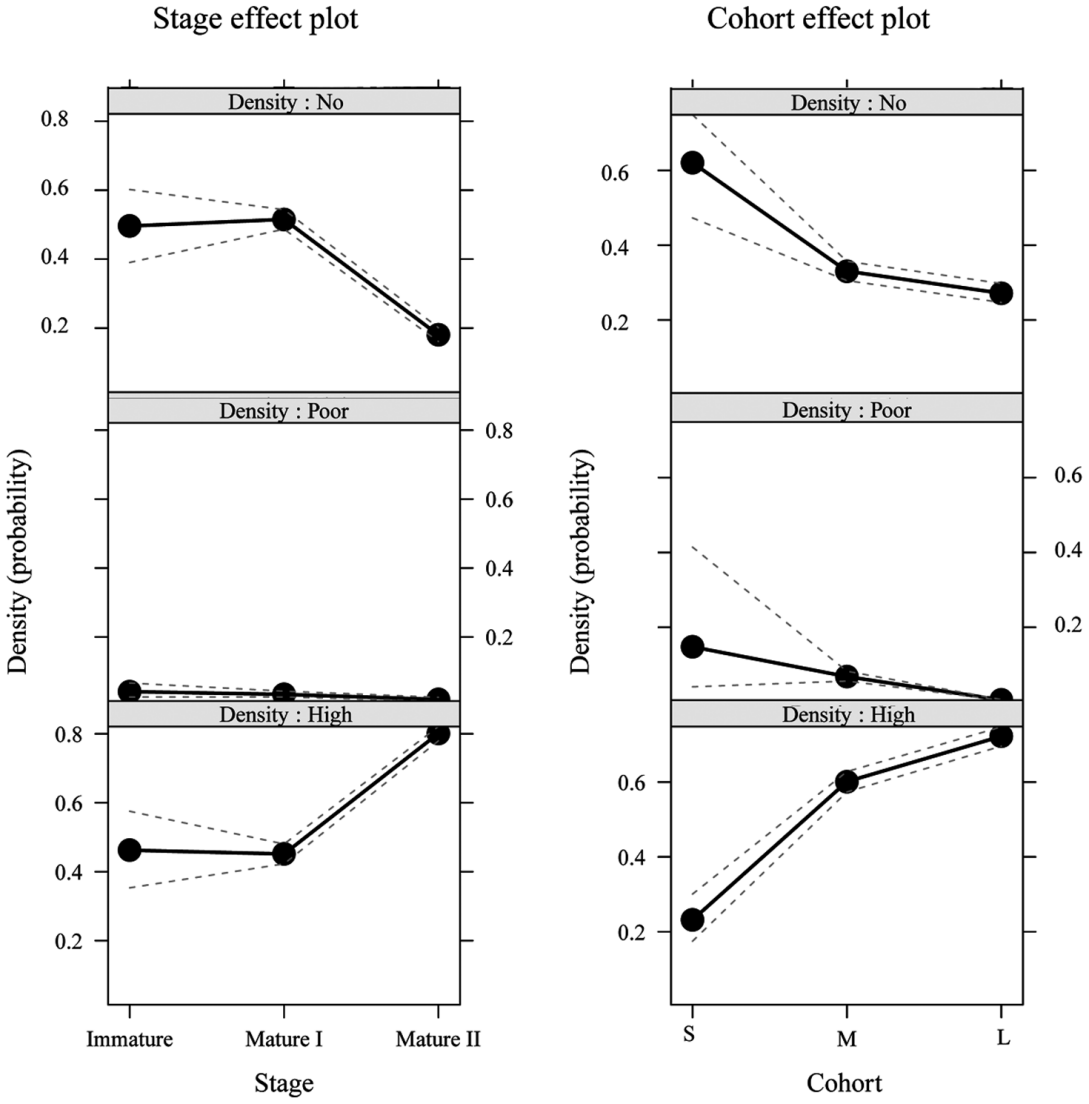
Main effect plots of *Wolbachia* infected oocyte proportion in the course of ovary maturation and oocyte development. The Y axis is labelled on the logit scale of the predictor (i.e. the probability scale of the response). The multinomial logistic regression showed that the proportion of *Wolbachia* infected oocytes per ovary depended on both the oocyte cohorts (*P*<2.2e−16) and the ovary maturation stage (*P*<2.2e−16). The proportion of infected oocytes remains stable between Immature and Mature I ovaries but increases between Mature I and Mature II ovaries. The proportion of infected oocytes increases throughout oocyte development.

**Figure 5:**
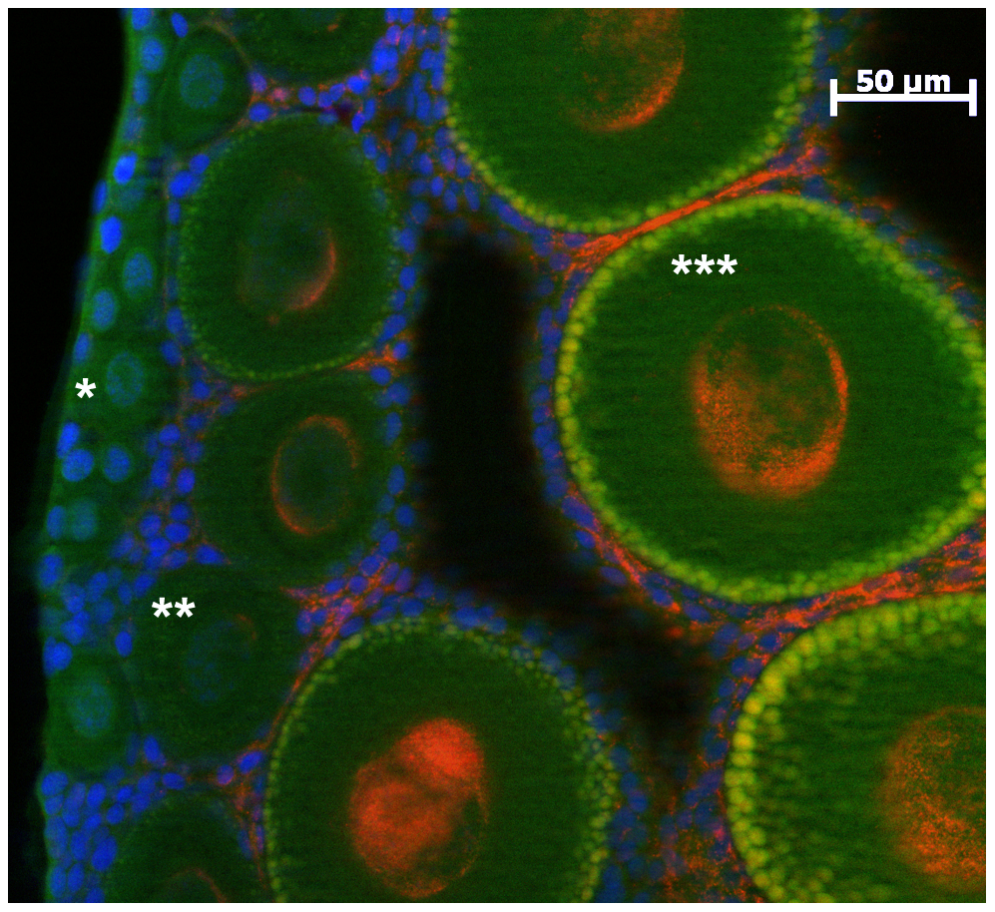
Close-up on FISH detection of *Wolbachia* in infected ovaries. *Wolbachia* appear in red and mostly cluster around the nucleus (blue). The yellow patterns at the periphery of some oocytes correspond to a type of vesicle which content autofluoresces both in red and green. In this Mature I ovary the germarium is on the left side, then some Small uninfected oocytes (e.g. *), and Medium size oocytes with representatives from the category “poorly infected” (**) and highly infected (***). Red: *Wolbachia* FISH probe W1,2-Cy3, green: phalloidin, blue: DAPI.

We determined the *Wolbachia* infection status of each oocyte of the 13 ovaries (Fig. 3). A multinomial logistic regression showed that the proportion of *Wolbachia* infected oocytes per ovary depended on both the oocyte cohorts (*P*<2.2e−16) and the ovary maturation stage (*P*<2.2e−16) (Fig. 4). Among infected oocytes, we were able to discriminate highly infected from poorly infected oocytes, i.e. oocytes presenting unusually low numbers of *Wolbachia* (e.g. Fig. 5). The latter were found at every ovary maturation stage and in every oocyte cohort. However their proportion decreased both in the course of ovary maturation (17.3% to 10.8% to 5.7% of the total number of oocytes) and oocyte development (14.2% to 6.9% to 0.3% of the total number of oocytes). Their proportion was very low and thus they had only a minor effect on the global infected-uninfected ratio. Therefore, for the following analyses “infected oocytes” stands for both highly and poorly infected oocytes.

**Figure 6:**
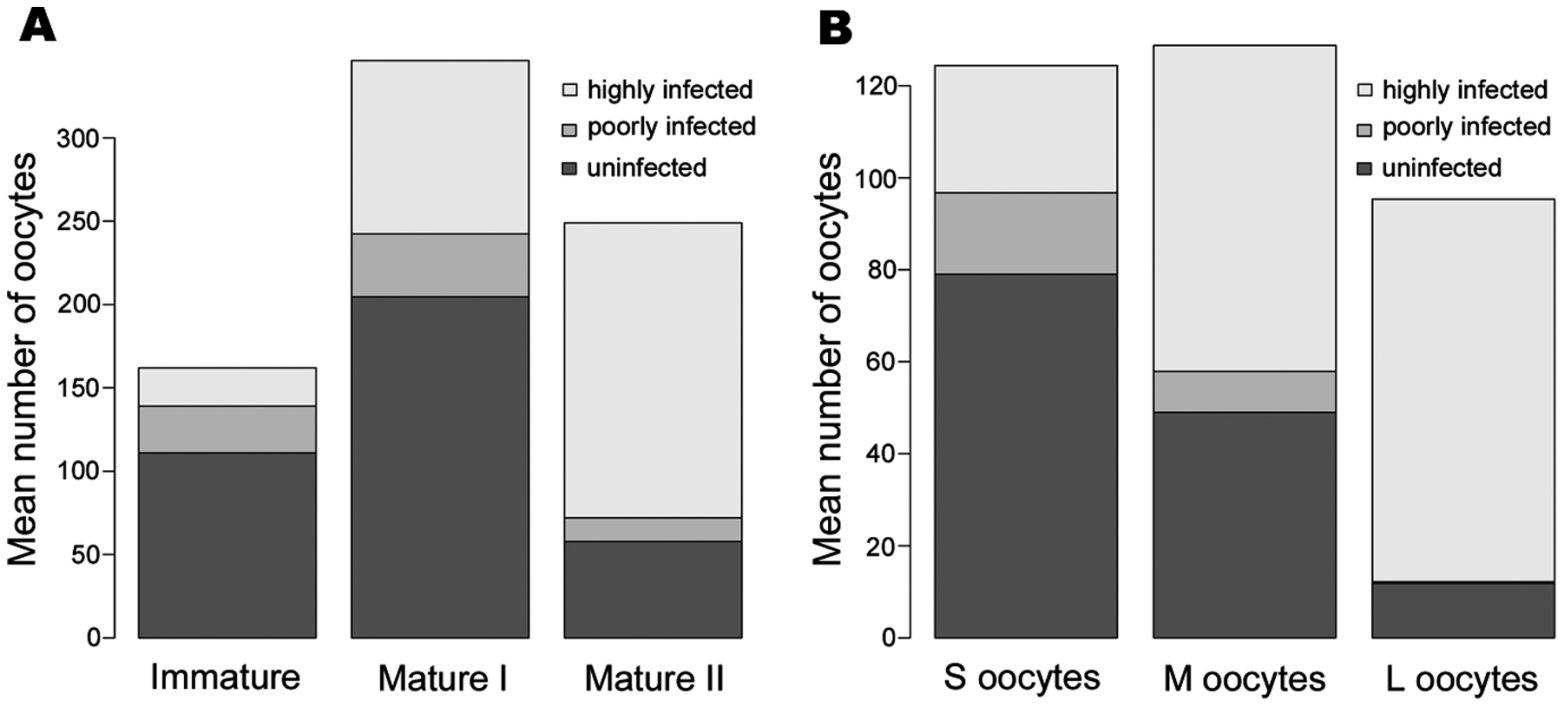
Average number of uninfected and infected (poorly and highly infected) oocytes per ovary for each ovary maturation stage (A) and in each oocyte developmental category (B). As ovaries mature (A), the total number of oocytes increases between the first two maturation stages and decreases between Mature I and Mature II. The numbers of uninfected and poorly infected oocytes follow the same pattern, whereas in contrast, the number of infected oocytes per ovary rises throughout ovary maturation. Again, as oocytes develop (B), the numbers of uninfected and poorly infected oocytes decrease while the number of infected oocytes increases between S and M categories only. Oocyte numberings were recorded from (**A**) one Immature ovary, six Mature I and six Mature II ovaries, (**B**) 13 ovaries containing S oocytes (all maturation stages), 12 ovaries containing M oocytes (Mature I and Mature II ovaries), and six ovaries containing L oocytes (Mature II ovaries). Standard errors are presented in the main text.

The maximum number of oocytes was observed in Mature I ovary maturation stage (mean ± s.e. per ovary: 346.3±35.5, Fig. 6A). Between Immature and Mature I ovary maturation stages, oocyte numbers doubled (from 162 to 346.3) but Mature II ovaries contained 28% less oocytes (mean ± s.e. per ovary: 249±18.9) than Mature I ovaries. This diminution of oocyte numbers between Mature I and Mature II ovaries seemed to concern uninfected oocytes only (mean number of uninfected oocytes  = 111 in Immature, 204.7±45.2 in Mature I, 57.8±8.6 in Mature II ovaries). In parallel, the number of oocytes infected by *Wolbachia* increased during ovary maturation (mean number of infected oocytes  = 51 in Immature, 141.7±26.9 in Mature I, 191.2±18.4 in Mature II ovaries). Consequently the proportion of infected oocytes became predominant in Mature II ovaries (multinomial logistic model: Stage effect plot, Fig. 4). It increased from 31.5% in Immature to 40.9% in Mature I, and to 76.8% in Mature II ovaries.

Across the 3734 oocytes recorded from the 13 ovaries, the proportion of *Wolbachia* infected oocytes increased with oocyte development (multinomial logistic model: Cohort effect plot, Fig. 4): 36.5% for the S cohort, 61.9% for the M cohort and 87.6% for the L cohort (Fig. 6A). When considering Small and Medium size oocytes, the proportion of infected oocytes rose due to both a decline of the number of uninfected oocytes and an increase of the number of infected oocytes. When considering Medium and Large size oocytes, there was a huge depletion of oocytes uninfected by *Wolbachia* (mean per ovary: 49±14.6 s.e. to 11.8±4.6 s.e.) whereas the number of infected oocytes remained constant (mean per ovary: 79.8±17.2 s.e. and 83.5±18.9 s.e.) (Fig. 6B).

#### *Wolbachia* infection patterns

In oocytes, the *Wolbachia* clustered as a crown around the nucleus (visible as dots of decondensed chromatin as expected with a meiotic prophase), without wrapping round it entirely, although the crown was not a simple band either (Fig. 3A and 5). Indeed, in most cases the bacteria bundled on one side as a mass, sometimes as two or three. The same pattern was found in poorly infected oocytes (Fig. 5). The masses in different oocytes were not facing the same direction, nor were they oriented along a maturation gradient within the ovary. The occasional single bacteria observed at the periphery of the oocytes were spherical. The follicle cells were infected as well, in a pattern that will be described below. So far no transmission figures of *Wolbachia* between cells were observed in Transmission Electron Microscopy (data not shown). However we observed, in one FISH sampling, many patterns that suggest possibilities of transfer via the follicle cells (Figures S1–S4).

**Figure 7:**
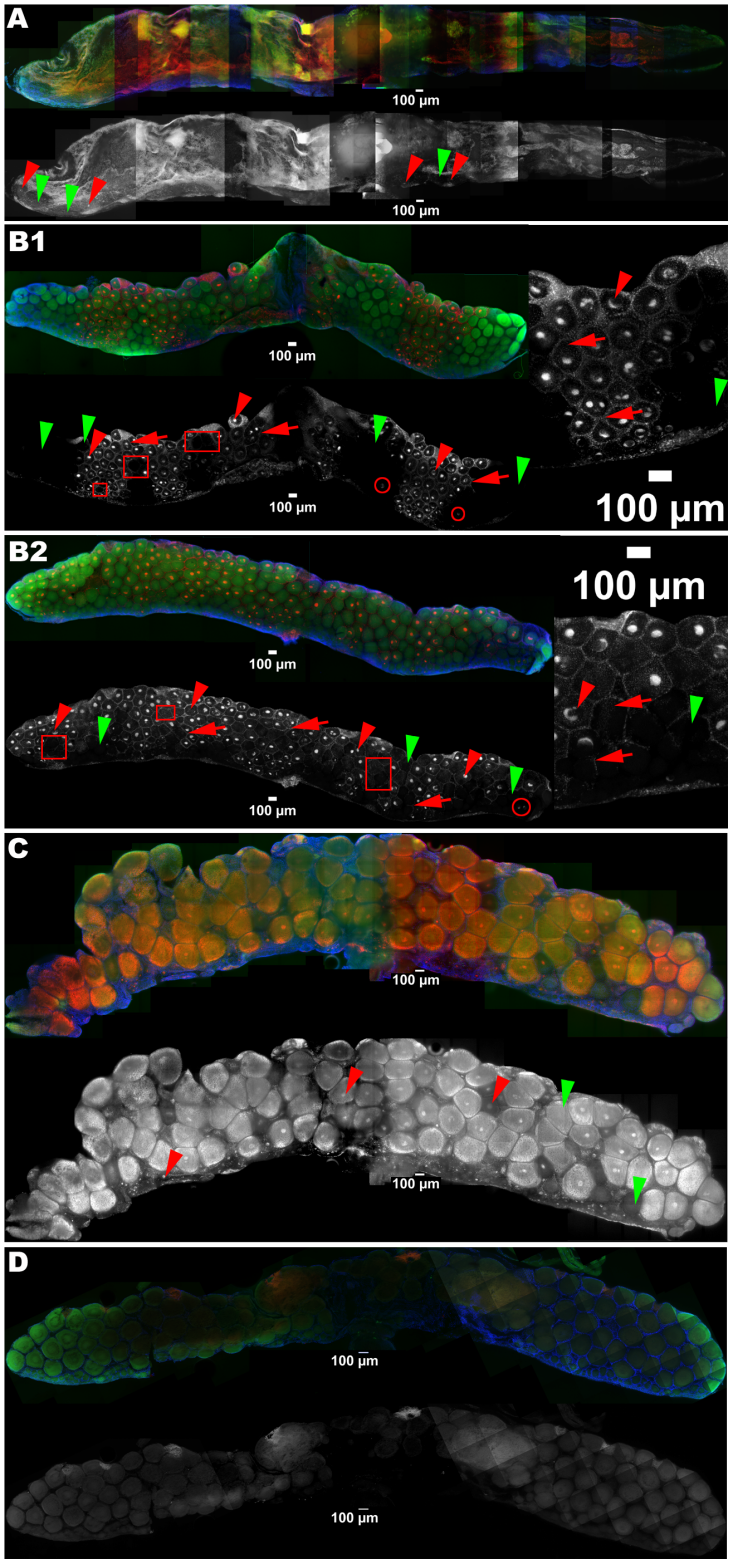
Average intensity projections of ovaries: *Wolbachia* (red and grayscale) detection in infected ovaries at different maturation stages: Immature (A), Mature I (B 1 and 2), Mature II (C), *versus* control uninfected ovary (D). The germaria are at the bottom, the scale bars placed in front of the oviduct insertion. **A**: Oocytes are only present along the thin band of the germarium (30.5% infected; infected oocytes: e.g. red arrowheads; uninfected ones: e.g. green arrowheads). **B**: *Wolbachia* are present as a mass near the nucleus of oocytes of all sizes (e.g. red arrowheads) and their follicle cells (infection there appears as a network surrounding the oocytes, e.g. red arrows), though many oocytes remained uncolonized (e.g. green arrowheads), especially the smaller ones. In the B1 ovary the colonized oocytes are roughly arranged as triangles, one tip stemming from the germarium side and expanding to the mature side (51% of infection), while the pattern in B2 (67% of infection) is more representative of this category, in so far as the colonization is not so linear. Also some colonized oocytes are not surrounded by infected follicle cells (e.g. red circles) and vice versa (e.g. red squares). **C**: Mature II is the most advanced stage in maturation (infected oocyte: e.g. red arrowhead; uninfected oocyte: e.g. green arrowhead). In addition to the *Wolbachia* signal the inside of the oocytes is filled with autofluorescing material, probably vitellogenin. **D**: Uninfected ovary. Red and grayscale: *Wolbachia* FISH probe W1,2-Cy3, green: phalloidin, blue: DAPI.

The Immature ovary (Fig. 7A) was almost empty and it contained only S oocytes. There were only a few infection foci in the germarium and even at times, they were not contiguous to infected areas, that is infected oocytes were not necessarily in the vicinity of an infected focus in the germarium.

In the six Mature I ovaries, both S and M oocyte categories contributed equally to the total population of oocytes. Uninfected oocytes tended to appear in clusters: whole areas of the ovaries remained uninfected. In general infected oocytes had no apparent distribution patterns although sometimes (Fig. 7B1), they were roughly arranged as triangles, one tip stemming from the germarium side and expanding to the mature side. Here, infected maturing oocytes seemed to proliferate from some patches in the germarium. The germarium was barely distinguishable and was sometimes broken up into a few foci, as inferred from the location of the smallest oocytes, with or without *Wolbachia*. The germarium was otherwise lined with FISH signals of variable densities. Most of these signals were *Wolbachia*, even if some artefacts were found in these areas in the negative controls. Sometimes (Fig. 7B1), the pattern of uninfected follicle cells matched almost perfectly that of uninfected oocytes, the network of infected follicle cells not expanding inside uninfected oocyte areas. However, for the other ovaries the infection was not that linear (e.g. Fig. 7B2, insert): some uninfected oocytes were surrounded by infected follicle cells and vice versa. Often, at the border of uninfected areas, the set of follicle cells surrounding one oocyte was only partly infected.

#### Control procedures

In the sub-samplings within ovaries (10 μm gap between optical slices), oocytes with a diameter below 30 μm were discarded because we had no random control full-depth scans corresponding to this oocyte diameter and attributing an infection status was therefore haphazard. Regarding the infection status, matching the oocytes of the sub-sampling to the control full-depth scans revealed very low rates of false positive and false negative (both 0.89%, i.e. one oocyte in the sub-sampling of 115 oocytes).

Only some aspecific signals in the *Wolbachia* channel were recorded from the ovaries of uninfected animals (Fig. 3B & 7D). They occurred mostly at two locations: as clumps outside of some of the most mature oocytes, corresponding to reported degrading areas, and as dots on the outermost side of the germarium area. None was observed inside oocytes.

**Figure 8:**
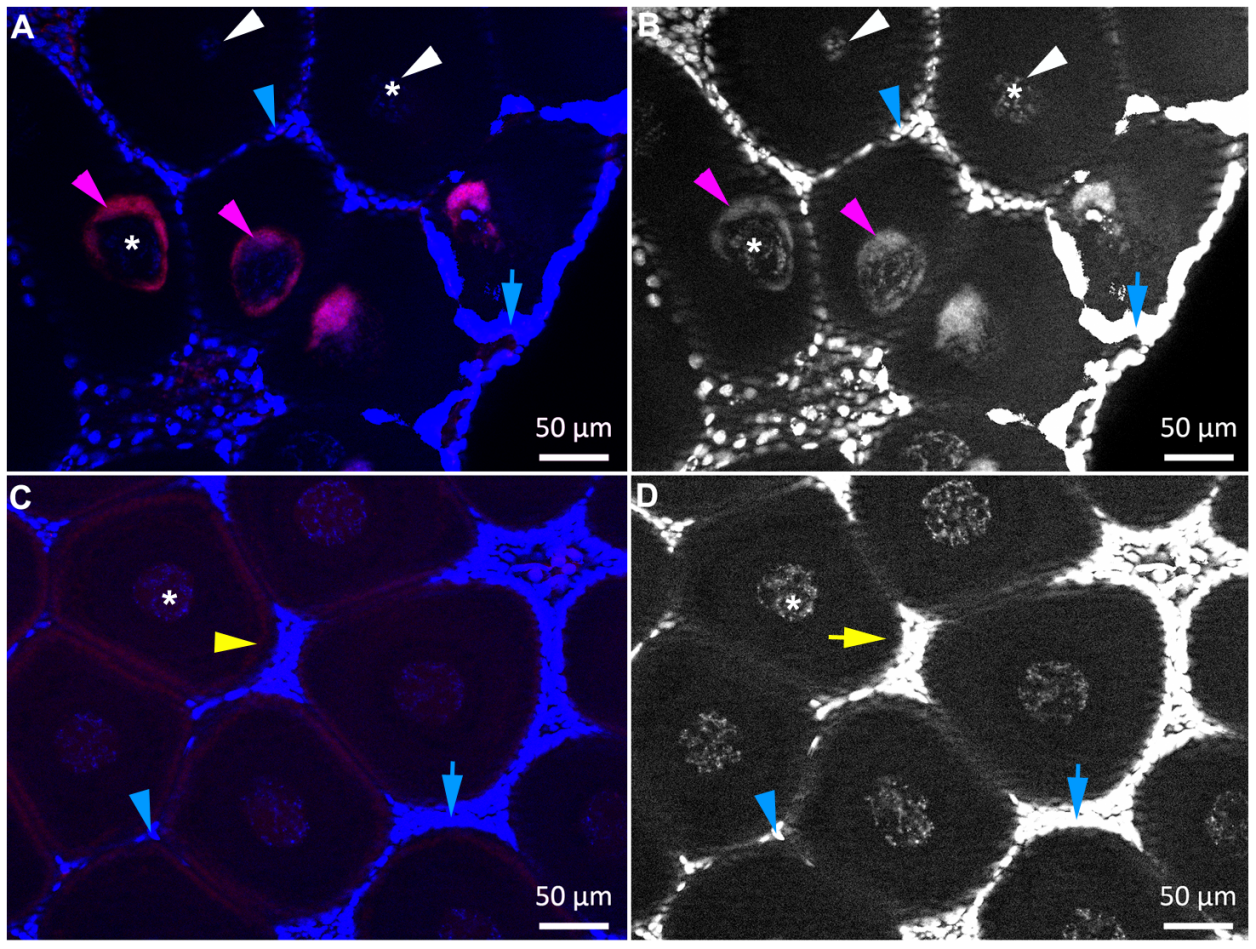
*Wolbachia* co-detection with DAPI and FISH in infected (A, B) versus control, uninfected ovaries (C, D). *Wolbachia* appear in purple (e.g. purple arrowheads). The DAPI staining (blue or grayscale) of oocyte nuclei (e.g. *) and *Wolbachia* is lower than the fluorescence emitted by follicle cells nuclei (e.g. blue arrowheads): its observation requires overexposing the nuclei of the follicle cells (e.g. blue arrows). In infected females (A, B) *Wolbachia* FISH and DAPI signals are detected around the nuclei in infected oocytes (e.g. purple arrowheads) while in uninfected oocytes only the nuclei are labelled (white arrowheads). In uninfected females only background was observed with the FISH probe (C: e.g. yellow arrowheads) which did not co-localize with DAPI staining (D: e.g. yellow arrow). Red: *Wolbachia* FISH probe W1,2-Cy3, blue and grayscale: DAPI.

Four infected and four uninfected ovaries were treated in tandem and alternated during the whole experiment, including imaging, to ensure that the absence of signal in oocytes from uninfected ovaries was not due to a failure of the FISH approach. *Wolbachia* signals were observed in the infected ovaries with the *Wolbachia* probe (Fig. 8A) and background staining was observed in the uninfected ovaries (Fig. 8C).

In addition in this sampling we checked that the *Wolbachia* probe co-localised with DAPI labelling of the bacteria inside oocytes (Fig. 8A and B). However revealing this co-staining required adjusting the imaging parameters manually for each image, and thus was not compatible with the automated imaging of whole ovaries performed above. The nuclei of the follicle cells emitted a much stronger fluorescence than the oocyte nucleus and *Wolbachia*, and this fluorescence increased when they were numerous, especially near the germarium. Imaging the oocyte nucleus and the *Wolbachia* was achieved at the cost of overexposing the nuclei of the follicle cells, which resulted in blurred blue halos surrounding the oocytes (Fig 8A and B, rightside), masking those closest to the germarium (data not shown).

## Discussion

We characterized the infection pattern of the feminizing strain *w*VulC in *Armadillidium vulgare* ovaries with Fluorescence *in situ* Hybridization to gain insight on the distribution of the bacteria at the tissue level. Unexpectedly, we observed recurrently, within the same lineages and among females belonging to the same broods, a high proportion of uninfected oocytes and whole areas lacking *Wolbachia* in ovaries that were beginning to mature while the mature ovaries in sibling females were largely colonized. Oocyte cohort analyses to infer the maturation stage confirmed that the number of *Wolbachia* infected oocytes increased throughout the reproductive cycle in *A. vulgare*, in the course of both oocyte development and ovary maturation. Among infected oocytes, we found only a very low proportion of poorly infected ones which decreased anyway in the course of both ovary maturation and oocyte development. Such a poor infection could represent either a failed infection or a transitory status before reinfection or *Wolbachia* multiplication. In parallel, observations of the infection patterns in ovaries revealed that the germarium, where the stem cells of the oocytes and of the follicle cells are both located, exhibited a scattered, poor infection. Consequently, we suspect other phenomena than acquisition through the germarium to be at the origin of *Wolbachia* enrichments.

The proportion of infected oocytes increased during the reproductive cycle to reach the known transmission rate to the progeny (∼82% [14]) in the most mature ovaries studied here (Mature II ovary stage) in the L cohort of oocytes (87.6%), those that will be spawned. A variation of the *Wolbachia* infection pattern between the reproductive cells of the same individual has been reported occasionally in insect and nematode models. In the nematode *Brugia malayi*, Fischer et al. [13] show the absence of *Wolbachia* in the young adult developing ovaries, using FISH and immunolocalization methods. In a *D. simulans* strain they consider as a natural variant, Casper-Lindley et al. [23] observe wholly uninfected egg chambers among infected ones, within the same ovariole, although they did not detect any uninfected offspring. Even more strikingly, in *B. malayi*, the situation can be as extreme as a young female having one developing infected ovary even though the other is *Wolbachia*-free [13]. In this nematode, *Wolbachia* is absent from the germline precursor at the end of embryogenesis, so *Wolbachia* is obligatory acquired secondarily through adjacent lateral chords. In *A. vulgare*, the lack of *Wolbachia* in many oocytes was neither accidental nor the result of natural variants since the multinomial logistic regression related the variation of the infection rate across individuals to the ovary maturation process.

We observed that in *A. vulgare* the germarium itself was far from fully infected, as in some ovaries whole areas in the margin clearly lacked *Wolbachia*. When we inferred the germarium location otherwise from the fanning out of very small oocytes, we observed many uninfected foci. This was especially obvious for the Immature ovary, where the layout was easier to inspect owing to the low number of oocytes. Such a low initial infection rate (i.e. 31.5% of infected oocytes) alone cannot account for an 82% transmission to the progeny and a sustainable infection over many generations. In addition, a primordial infection of oogonia, without any enrichment mechanisms, could not be sufficient to explain the increase of the number of infected oocytes in the course of both ovary and oocyte maturation. We propose that the compensation for the low infection of the germarium may result from two complementary mechanisms regarding the transmission strategies of *Wolbachia*: i) *Wolbachia* were acquired by the ovaries from somatic tissues and ii) the oocytes containing *Wolbachia* were selected.

A secondary infection from a somatic reservoir seems likely, since it is a conserved trait among *Wolbachia* strains [6], [7], [13]. In *Drosophila*, the entry points to facilitate the infection of the oogonia were located in their vicinity, in the germarium [4]. In *A. vulgare*, such an early location would only supply more infection foci in the germarium and would not account for the increase of *Wolbachia* infection among oocytes during the ovarian cycle. A possible *Wolbachia* enrichment in oocytes would more probably occur after oocytes left the germarium stage. Indeed, Fischer et al. [13] have shown that *Wolbachia* coming from a reservoir organ (lateral chords) can infect oocytes of young *B. malayi* roundworms during their development in the maturation zone of the ovary. Actually, the consequent increase in *Wolbachia* infection we observed in *A. vulgare* occurred in the course of ovary maturation, being stronger between Mature I and Mature II ovaries, and oocyte development, especially between S and M oocytes. While the number of oocytes in these categories was the same, the infection ratio really swapped from S to M, as if uninfected oocytes became infected. We believe there were too many M infected oocytes for it to be the sole consequence of the maturation of the S infected oocytes.

Cytoplasmic dumping, proposed as a means to rescue failed infection in oocytes [24], actually happens during late vitellogenesis in *Drosophila*. However woodlice have neither nurse cells nor cytoplasmic bridges that connect oocytes with other cells. Other endosymbionts are transmitted during vitellogenesis as well, via the follicle cells (microsporidia in *Gammarus duebni*, [25]) or passing through them from the haemolymph to reach the oocytes (Yeast-Like Symbionts in *Metcalfa pruinosa* and *Conomelus anceps* [26], *Spiroplasma poulsonii* in *D. melanogaster* [27]). Toomey et al. [6] recently proposed that the follicle cells pass along *Wolbachia* to the oocytes as they mature, as a cell-to-cell channeling of the infection from the SSCN of *Drosophila*. In our case, such transmitters are all the more likely considering that at the transition zones between infected and *Wolbachia*-free areas in ovaries, many uninfected oocytes were surrounded by infected follicle cells. In addition many follicles (containing either an infected or uninfected oocyte) were partly infected in so far as, for example, one half was colonized and the other one was not.

*Wolbachia* can infect *de novo* woodlice ovaries via injection of an infected ovary suspension [28], [29], after a contact with infected haemolymph [30], or by transplantation of infected tissues [28], [29], [31], confirming that a cell-to-cell passage is possible. We will now investigate the infection pattern after *Wolbachia* transinfection, the method used by Frydman et al. [4] to highlight secondary acquisition sites in *D. melanogaster* ovaries.

In addition, we suspect infected oocytes were selected by escaping oosorption to secure *Wolbachia* secondary acquisition. Oosorption is a continuous process in isopod ovaries and involves apoptosis [32]. Some large oocytes are lost and not all will mature into eggs to be laid in the marsupial pouch [32]. Additionally, Besse [18] notes that younger oocytes of the terrestrial isopod *Porcellio dilatatus* can also degenerate as ovaries mature. We noted a decrease of the total oocyte number between Mature I and Mature II ovaries that only affected uninfected oocytes. This suggests a possible selection of infected oocytes via, for example, a protection against apoptosis during the transition between Mature I and II ovary stages, which would finally contribute to raise the prevalence of infected oocytes to 87.6% by eliminating uninfected ones. This selection mechanism would fit Werren's assumption [1] regarding the so-called post-segregation killing, at the passing of a cell death checkpoint. He adapted this concept, that otherwise applies to genetic elements, to a population of germinal stem cells, where a heritable symbiont can be lost stochastically during proliferation. He further proposed that the failure of *Asobara tabida* to produce eggs after removing *Wolbachia* could be the result of the death of the uninfected germ cells. *Wolbachia* protection against apoptosis in *A. tabida* ovaries was confirmed by Pannebakker et al. [33] who actually located apoptosis phenomena in nurse cells at mid-oogenesis, provoking the breakdown of whole egg chambers. This is a typical cell death checkpoint in *Drosophila* to eliminate oocytes following inadequate environmental conditions [34]. In *A. vulgare*, the huge decrease of oocytes between Mature I and Mature II ovaries (mid-oogenesis) corroborates such a kind of checkpoint. In *D. mauritiana* Fast et al. [5] showed that *Wolbachia* infection induces a decrease in programmed cell death in the germarium which, concomitantly with an increase in stem cell mitotic activity, leads to egg overproduction. In nematodes, following antibiotic curing of *Wolbachia* apoptosis not only increases in ovaries, but also massively affects intrauterine embryos and larvae [35]. In the latter, even cells that would not have harboured *Wolbachia* undergo apoptosis: therefore Landmann et al. (2011) infer an indirect, non cell-autonomous mechanism, probably related to a defect in trophic supplies from the hypodermal chords due to *Wolbachia* depletion. In *A. vulgare*, the decrease of uninfected oocytes numbers suggests in contrast a cell-autonomous effect, i.e. that *Wolbachia* presence in an oocyte would directly prevent its elimination. We will look for apoptosis in oocytes not containing *Wolbachia* to support our assumption.

In summary, we have highlighted the plasticity of *Wolbachia* infection in *A. vulgare*, particularly in relation with the host ovary maturation cycle. Whatever the mechanism, we have shown an enrichment of *Wolbachia* in the course of oocyte and ovary maturation, eventually reaching the known transmission rate to offspring. We hypothesize *Wolbachia* infect oocytes when maturation has already begun like last minute passengers, and/or benefit from a favorable oocyte selection like priority travelers (Fig. 1). While protection from apoptosis is well documented for *Wolbachia* [5], [34], [36], the secondary infection scenario raises several issues not considered thus far. Obviously transinfection experiments demonstrate *Wolbachia* perform well in cell-to-cell transmission, and this is the route used in *B. malayi* larvae to reach the ovaries from the adjacent lateral chords [12] and in *Drosophila* from the Niches [6]. In *A. vulgare*, a more distant reservoir has been proposed, requiring mobile vectors, infected haemocytes, to taxi *Wolbachia* [9], [28], [30]. There is also the question of the cues that trigger such a migration, which seem to be connected to host physiology (development in *B. malayi* and here, ovary maturation in *A. vulgare*). Exploring these new fields may also clarify the factors that determine the failure or success of *Wolbachia* colonizing a new host.

## Supporting Information

Figure S1 **Potential transmission figures of** ***Wolbachia*** **from the follicle cells to the oocytes.** Red and gray scale: *Wolbachia* FISH probe W1,2-Cy3, green: phalloidin, blue: DAPI. In seven sister females sampled during the same period, we observed many patterns of cellular infection suggesting that oocytes can acquire *Wolbachia* from the follicle cells. These females had synchronized maturation cycles since they were reared together. The pattern of infection seemed to be continuous between the follicle cells and the oocytes (red arrows). The hallmark is the elongated form of many *Wolbachia* that may correspond to single cells or chains of cells (red arrowheads). In some oocytes, the *Wolbachia* formed a diffuse crown around the nucleus instead of a compact one as we usually observe, and several others were located at the periphery, very close to the follicle cells (A and B, arrows). Strickingly in (B) the follicle cells surrounding the oocyte were uninfected, except for those in the continuity of a series of follicle cells containing *Wolbachia* oriented as a line (arrowheads in B); part of an infected area is visible in the lower left corner. Alternatively instead of a crown we observed only diffuse clouds of *Wolbachia* at the periphery (C). This phenomenon may correspond to a natural variant of *Wolbachia*. However, a subsequent sampling of other sisters only yielded several observations of the same type, then no more (data not shown). Therefore we suspect it is more likely that we happened on a transitional phenomenon. Whatever the reason for this phenomenon, the images suggest possibilities of transfer via the follicle cells which needs to be demonstrated by TEM.
(TIF)

Figure S2 **3D reconstructions from the image presented in S1A, 0.6 μm between slices.** Red: *Wolbachia* FISH probe W1,2-Cy3, green: phalloidin, blue: DAPI.
(MOV)

Figure S3 **3D reconstructions from the image presented in S1B, 0.6 μm between slices.** Red: *Wolbachia* FISH probe W1,2-Cy3, green: phalloidin, blue: DAPI.
(MOV)

Figure S4 **3D reconstructions from the image presented in S1C, 0.6 μm between slices.** Red: *Wolbachia* FISH probe W1,2-Cy3, green: phalloidin, blue: DAPI.
(MOV)

## References

[1] Werren JH (2005) Heritable Microorganisms and Reproductive Parasitism. In: J S, editor. Microbial phylogeny and evolution: concepts and controversies: Oxford University Press. pp. 290–315.

[2] Veneti Z, Clark ME, Karr TL, Savakis C, Bourtzis K (2004) Heads or tails: host-parasite interactions in the *Drosophila*-*Wolbachia* system. Appl Environ Microbiol 70: 5366–5372. 15345422 10.1128/AEM.70.9.5366-5372.2004 PMC520876

[3] Kose H, Karr TL (1995) Organization of *Wolbachia* pipientis in the *Drosophila* fertilized egg and embryo revealed by an anti-*Wolbachia* monoclonal antibody. Mech Dev 51: 275–288. 7547474 10.1016/0925-4773(95)00372-x

[4] Frydman HM, Li JM, Robson DN, Wieschaus E (2006) Somatic stem cell niche tropism in *Wolbachia* . Nature 441: 509–512. 16724067 10.1038/nature04756

[5] Fast EM, Toomey ME, Panaram K, Desjardins D, Kolaczyk ED, et al (2011) *Wolbachia* enhance *Drosophila* stem cell proliferation and target the germline stem cell niche. Science 334: 990–992. 22021671 10.1126/science.1209609 PMC4030408

[6] Toomey ME, Panaram K, Fast EM, Beatty C, Frydman HM (2013) Evolutionarily conserved *Wolbachia*-encoded factors control pattern of stem-cell niche tropism in Drosophila ovaries and favor infection. Proc Natl Acad Sci U S A 110: 10788–10793. 23744038 10.1073/pnas.1301524110 PMC3696799

[7] Sacchi L, Genchi M, Clementi E, Negri I, Alma A, et al (2010) Bacteriocyte-like cells harbour *Wolbachia* in the ovary of *Drosophila melanogaster* (Insecta, Diptera) and *Zyginidia pullula* (Insecta, Hemiptera). Tissue Cell 42: 328–333. 20817243 10.1016/j.tice.2010.07.009

[8] Albertson R, Casper-Lindley C, Cao J, Tram U, Sullivan W (2009) Symmetric and asymmetric mitotic segregation patterns influence *Wolbachia* distribution in host somatic tissue. J Cell Sci 122: 4570–4583. 19934219 10.1242/jcs.054981 PMC2787466

[9] Huynh JR, St Johnston D (2004) The origin of asymmetry: early polarisation of the *Drosophila* germline cyst and oocyte. Curr Biol 14: R438–449. 15182695 10.1016/j.cub.2004.05.040

[10] Serbus LR, Casper-Lindley C, Landmann F, Sullivan W (2008) The genetics and cell biology of *Wolbachia*-host interactions. Annu Rev Genet 42: 683–707. 18713031 10.1146/annurev.genet.41.110306.130354

[11] Landmann F, Foster JM, Slatko B, Sullivan W (2010) Asymmetric *Wolbachia* segregation during early *Brugia malayi* embryogenesis determines its distribution in adult host tissues. PLoS Negl Trop Dis 4: e758. 20689574 10.1371/journal.pntd.0000758 PMC2910707

[12] Landmann F, Bain O, Martin C, Uni S, Taylor MJ, et al (2012) Both asymmetric mitotic segregation and cell-to-cell invasion are required for stable germline transmission of *Wolbachia* in filarial nematodes. Biol Open 1: 536–547. 23213446 10.1242/bio.2012737 PMC3509449

[13] Fischer K, Beatty WL, Jiang D, Weil GJ, Fischer PU (2011) Tissue and stage-specific distribution of *Wolbachia* in *Brugia malayi* . PLoS Negl Trop Dis 5: e1174. 21629728 10.1371/journal.pntd.0001174 PMC3101188

[14] Cordaux R, Michel-Salzat A, Frelon-Raimond M, Rigaud T, Bouchon D (2004) Evidence for a new feminizing *Wolbachia* strain in the isopod *Armadillidium vulgare*: evolutionary implications. Heredity (Edinb) 93: 78–84. 15138452 10.1038/sj.hdy.6800482

[15] Bouchon D, Rigaud T, Juchault P (1998) Evidence for widespread *Wolbachia* infection in isopod crustaceans: molecular identification and host feminization. Proc Biol Sci 265: 1081–1090. 9684374 10.1098/rspb.1998.0402 PMC1689171

[16] Martin G, Legrand P, Juchault JJ (1973) Mise en évidence d'un micro-organisme intracytoplasmique symbiote de l'Oniscoïde *Armadillidium vulgare* L. dont la présence accompagne l'intersexualité ou la féminisation totale des mâles génétiques de la lignée thélygène. Comptes Rendus de l'Académie des Sciences Paris 76: 2313–2316.

[17] Rousset F, Bouchon D, Pintureau B, Juchault P, Solignac M (1992) *Wolbachia* endosymbionts responsible for various alterations of sexuality in arthropods. Proc Biol Sci 250: 91–98. 1361987 10.1098/rspb.1992.0135

[18] Besse G (1976) Contribution à l'étude expérimentale de la physiologie sexuelle femelle chez les Crustacés Isopodes terrestres. PhD thesis. France. University of Poitiers.

[19] Souty-Grosset C (1984) Contribution à l'étude de la synthèse des constituants du vitellus protéique et de son contrôle humoral chez deux crustacés iopodes *Idotea balthica basteri* Audouin (Valvifere) et *Porcellio dilatatus* Brandt (Oniscoïde).

[20] Manz W, Amann R, Ludwig W, Vancanneyt M, Schleifer KH (1996) Application of a suite of 16S rRNA-specific oligonucleotide probes designed to investigate bacteria of the phylum cytophaga-flavobacter-bacteroides in the natural environment. Microbiology 142 (Pt 5): 1097–1106. 10.1099/13500872-142-5-1097 8704951

[21] Heddi A, Grenier AM, Khatchadourian C, Charles H, Nardon P (1999) Four intracellular genomes direct weevil biology: nuclear, mitochondrial, principal endosymbiont, and *Wolbachia* . Proc Natl Acad Sci U S A 96: 6814–6819. 10359795 10.1073/pnas.96.12.6814 PMC21998

[22] Chevalier F, Herbiniere-Gaboreau J, Bertaux J, Raimond M, Morel F, et al (2011) The immune cellular effectors of terrestrial isopod *Armadillidium vulgare*: meeting with their invaders, *Wolbachia* . PLoS ONE 6: e18531. 21533137 10.1371/journal.pone.0018531 PMC3080368

[23] Casper-Lindley C, Kimura S, Saxton DS, Essaw Y, Simpson I, et al (2011) Rapid fluorescence-based screening for *Wolbachia* endosymbionts in *Drosophila* germ line and somatic tissues. Appl Environ Microbiol 77: 4788–4794. 21622788 10.1128/AEM.00215-11 PMC3147364

[24] Ferree PM, Frydman HM, Li JM, Cao J, Wieschaus E, et al (2005) *Wolbachia* utilizes host microtubules and Dynein for anterior localization in the *Drosophila* oocyte. PLoS Pathog 1: e14. 16228015 10.1371/journal.ppat.0010014 PMC1253842

[25] Terry RS, Dunn AM, Smith JE (1997) Cellular distribution of a feminizing microsporidian parasite: a strategy for transovarial transmission. Parasitology 115 (Pt 2): 157–163. 10.1017/s0031182097001236 10190171

[26] Michalik A, Jankowska W, Szklarzewicz T (2009) Ultrastructure and transovarial transmission of endosymbiotic microorganisms in *Conomelus anceps* and *Metcalfa pruinosa* (Insecta, Hemiptera, Fulgoromorpha). Folia Biol (Krakow) 57: 131–137. 19777955 10.3409/fb57_3-4.131-137

[27] Herren JK, Paredes JC, Schupfer F, Lemaitre B (2013) Vertical transmission of *Drosophila* endosymbiont via cooption of the yolk transport and internalization machinery. MBio 4.. 10.1128/mBio.00532-12 PMC3585447 23462112

[28] Rigaud T, Souty-Grosset C, Raimond R, Mocquard JP, Juchault P (1991) Feminizing endocytobiosis in the terrestrial crustacean *Armadillidium vulgare* Latr. (Isopoda): recent acquisitions. *Endocytol Cell Res* 7: 259–273.

[29] Juchault P, Frelon-Raimond M, Bouchon D, Rigaud T (1994) New evidence for feminizing bacteria in terrestrial iopods: evolutionary implications. C R Acad Sci 317: 225–230.

[30] Rigaud T, Juchault P (1995) Success and failure of horizontal transfers of feminizing *Wolbachia* endosymbionts in woodlice. *J Evol Biol* 8: 249–255.

[31] Juchault P, Legrand JJ, Martin G (1974) Action interspécifique du facteur épigénétique féminisant responsable de la thélygénie et de l'intersexualité du crustacé *Armadillidium vulgare* (Isopode Oniscoïde). Annales d'Embryologie et de Morphogénèse 7: 265–276.

[32] Hornung E, Warburg MR (1994) Oosorption and oocyte loss in a terrestrial isopod under stressful conditions. Tissue Cell 26: 277–284. 18621271 10.1016/0040-8166(94)90102-3

[33] Pannebakker BA, Loppin B, Elemans CP, Humblot L, Vavre F (2007) Parasitic inhibition of cell death facilitates symbiosis. Proc Natl Acad Sci U S A 104: 213–215. 17190825 10.1073/pnas.0607845104 PMC1765438

[34] McCall K (2004) Eggs over easy: cell death in the Drosophila ovary. Dev Biol 274: 3–14. 15355784 10.1016/j.ydbio.2004.07.017

[35] Landmann F, Voronin D, Sullivan W, Taylor MJ (2011) Anti-filarial activity of antibiotic therapy due to extensive apoptosis after *Wolbachia* depletion from filarial nematodes. PLoS Pathog 7: e1002351. 22072969 10.1371/journal.ppat.1002351 PMC3207916

[36] Bazzocchi C, Comazzi S, Santoni R, Bandi C, Genchi C, et al (2007) *Wolbachia* surface protein (WSP) inhibits apoptosis in human neutrophils. Parasite Immunol 29: 73–79. 17241395 10.1111/j.1365-3024.2006.00915.x

