# Supplementary figures and images for: Wolbachia Infect Ovaries in the Course of Their Maturation: Last Minute Passengers and Priority Travellers?
Source: PLoS One. 2014 Apr 10;9(4):e94577. doi: 10.1371/journal.pone.0094577 (PMC3983217; doi:10.1371/journal.pone.0094577)

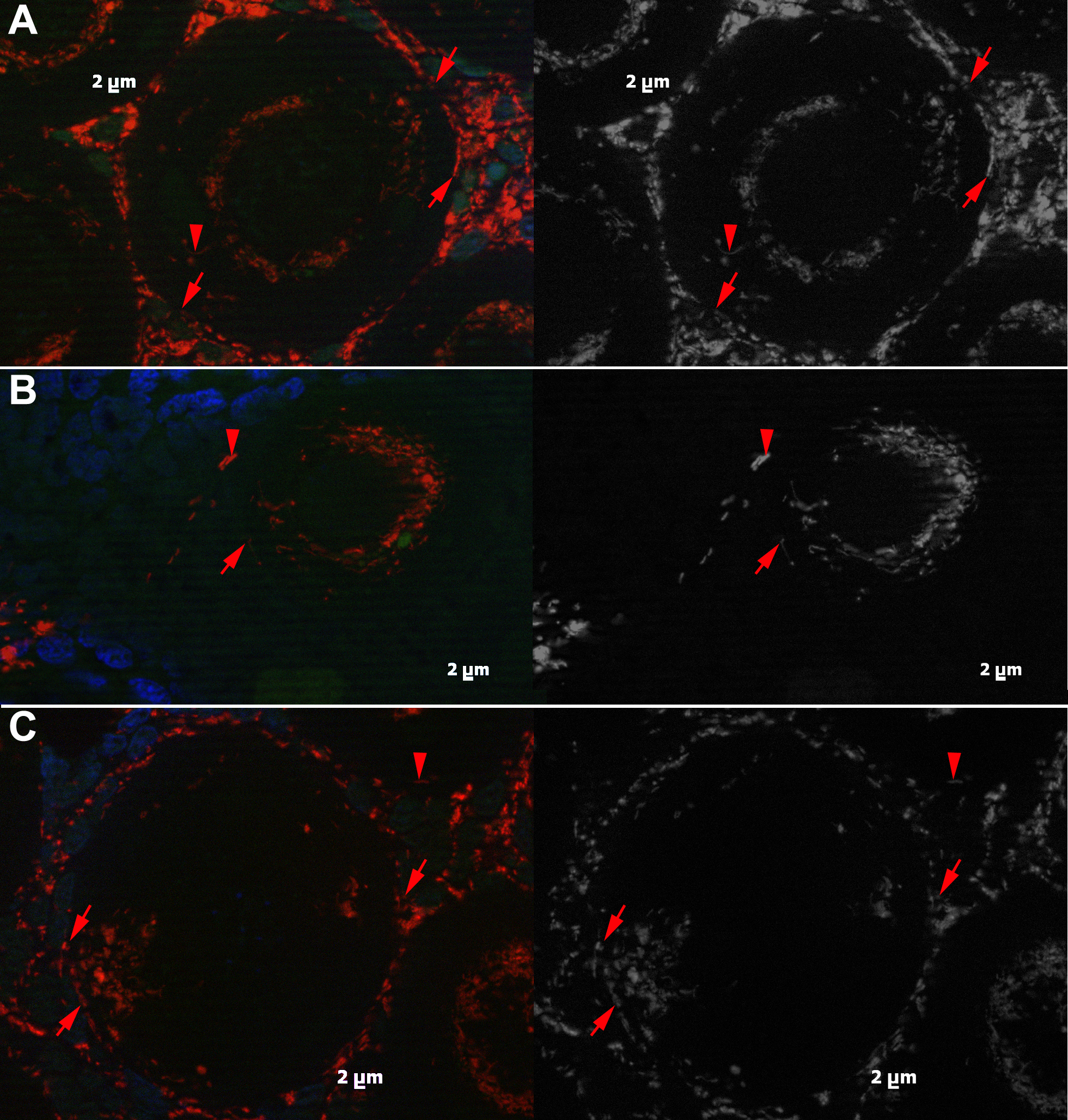

Supplement: Figure S1 — Potential transmission figures of Wolbachia from the follicle cells to the oocytes. Red and gray scale: Wolbachia FISH probe W1,2-Cy3, green: phalloidin, blue: DAPI. In seven sister females sampled during the same period, we observed many patterns of cellular infection suggesting that oocytes can acquire Wolbachia from the follicle cells. These females had synchronized maturation cycles since they were reared together. The pattern of infection seemed to be continuous between the follicle cells and the oocytes (red arrows). The hallmark is the elongated form of many Wolbachia that may correspond to single cells or chains of cells (red arrowheads). In some oocytes, the Wolbachia formed a diffuse crown around the nucleus instead of a compact one as we usually observe, and several others were located at the periphery, very close to the follicle cells (A and B, arrows). Strickingly in (B) the follicle cells surrounding the oocyte were uninfected, except for those in the continuity of a series of follicle cells containing Wolbachia oriented as a line (arrowheads in B); part of an infected area is visible in the lower left corner. Alternatively instead of a crown we observed only diffuse clouds of Wolbachia at the periphery (C). This phenomenon may correspond to a natural variant of Wolbachia. However, a subsequent sampling of other sisters only yielded several observations of the same type, then no more (data not shown). Therefore we suspect it is more likely that we happened on a transitional phenomenon. Whatever the reason for this phenomenon, the images suggest possibilities of transfer via the follicle cells which needs to be demonstrated by TEM. (TIF) [file pone.0094577.s001.tif]
